# Differential Inhibition by Cenobamate of Canonical Human Nav1.5 Ion Channels and Several Point Mutants

**DOI:** 10.3390/ijms26010358

**Published:** 2025-01-03

**Authors:** Teodor Asvadur Şulea, Sorin Draga, Maria Mernea, Alexandru Dan Corlan, Beatrice Mihaela Radu, Andrei-Jose Petrescu, Bogdan Amuzescu

**Affiliations:** 1 Department of Bioinformatics and Structural Biochemistry, Institute of Biochemistry of the Romanian Academy, Splaiul Independentei 296, 060031 Bucharest, Romania; teodor.sulea@biochim.ro (T.A.Ş.); andrei.petrescu@biochim.ro (A.-J.P.); 2 Biotehnos SA, Gorunului Str. 3-5, 075100 Otopeni, Romania; sor.draga@gmail.com; 3 Non-Governmental Research Organization Biologic, 14 Schitului Str., 032044 Bucharest, Romania; 4 Department of Anatomy, Animal Physiology and Biophysics, Faculty of Biology, University of Bucharest, Splaiul Independentei 91-95, 050095 Bucharest, Romania; beatrice.radu@bio.unibuc.ro (B.M.R.); bogdan@biologie.kappa.ro (B.A.); 5 Cardiology Research Unit, University and Emergency Hospital of Bucharest, Splaiul Independenței 169, 050098 Bucharest, Romania; alexandru@corlan.net

**Keywords:** cenobamate, cardiac voltage-dependent sodium channel, Nav1.5, structural model, molecular docking, molecular dynamics, binding free energy, MM-GBSA, MM-PBSA, point mutant

## Abstract

Cenobamate is a new and highly effective antiseizure compound used for the treatment of adults with focal onset seizures and particularly for epilepsy resistant to other antiepileptic drugs. It acts on multiple targets, as it is a positive allosteric activator of γ-aminobutyric acid type A (GABA_A_) receptors and an inhibitor of neuronal sodium channels, particularly of the late or persistent Na^+^ current. We recently evidenced the inhibitory effects of cenobamate on the peak and late current component of the human cardiac isoform hNav1.5. The determined apparent IC_50_ values of 87.6 µM (peak) and 46.5 µM (late current) are within a clinically relevant range of concentrations (the maximal plasma therapeutic effective concentration for a daily dose of 400 mg in humans is 170 µM). In this study, we built a 3D model of the canonical hNav1.5 channel (UniProt Q14524-1) in open conformation using AlphaFold2, embedded it in a DPPC lipid bilayer, corrected the residue protonation state (pH 7.2) with H++, and added 2 Na^+^ ions in the selectivity filter. By molecular docking, we found the cenobamate binding site in the central cavity. We identified 10-point mutant variants in the binding site region and explored them via docking and MD. Mutants N1462K/Y (rs1064795922, rs199473614) and M1765R (rs752476527) (by docking) and N932S (rs2061582195) (by MD) featured higher predicted affinity than wild-type.

## 1. Introduction

Voltage-dependent Na^+^ channels (Nav) are members of a superfamily of voltage-gated ion channels included in the chanome [1]. These are derived from primordial prokaryotic tetrameric K^+^ channels with two α-helical transmembrane (TM) domains per subunit (e.g., Kcsa [2,3]) by adding a 4TM helix voltage-sensing domain (VSD) [4,5]. Through consecutive gene duplication events, Nav channels and related voltage-dependent Ca^2+^ channels (Cav) present the sequences of all four homologous 6TM domains (I-IV) of the tetrameric channel assemblies on the same chain [6]. Beyond the main pore-forming α1 subunits, Nav channels feature four types of ancillary β subunits (β1–β4), with a single TM α-helix per subunit and a large antiparallel β-sheet immunoglobulin-like extracellular domain [1] interacting with the pore loops between α helices S5 and S6 of each domain of the main α1 subunit [7]. Due to the structural similarity of β subunits with cell adhesion molecules, they play a variety of roles in neurons, such as their involvement in neural cell proliferation, migration, neurite extension, myelination, and influence on neuronal firing by the regulation of Nav α subunits’ gene expression and trafficking, the modulation of gating events such as voltage-dependent activation, the coupling of activation with fast inactivation, and late current modes [8,9]. However, for the cardiomyocyte-specific Nav isoform (Nav1.5), such roles have not been proved [10].

As suggested by the classical studies included in the Hodgkin–Huxley model [11] and the gating current recordings of Clay M. Armstrong and Francisco Bezanilla [12], the S4 helix of the VSD features positively charged residues (R/K) at every third position and functions as a voltage sensor. This fact was confirmed after the cloning of a Nav α subunit gene [13] and subsequent charge-neutralizing point mutations within S4, leading to decreased voltage dependence of activation [14]. Mutagenesis studies have shown that the S4 voltage sensors of Nav domains I-III contribute to the activation gating current, while the S4 of domain IV is largely involved in fast voltage-dependent inactivation [15,16,17,18,19]. The fast “inactivation particle” effecting this state transition is formed by the highly conserved hydrophobic IFMT motif in the internal loop between domains III and IV [20,21,22,23]. Recent phototrapping experiments pointed to a lateral pore-adjacent binding site for this particle and an allosteric mechanism resulting in the pinching of the S6 inner gate [24]. Meanwhile, slow voltage-dependent inactivation is deemed to result from conformational changes in the region of the selectivity filter and external S6 helices, and their reversibility upon hyperpolarization is also slow compared to fast inactivation and recovery [25,26,27]. Nav channels feature a large number of serine/threonine phosporylation sites on their endodomain [28,29], as well as interaction motifs for a large number of regulatory proteins and cytoskeletal components [7,30].

A large number of studies have been performed on Nav channels to elucidate the molecular details of interaction with local anesthetics, antiseizure drugs, or antiarrhythmic medications [1]. Structural and mutagenesis studies identified several residues in the S6 helices of multiple domains participating in drug binding, upon drug entry into the central cavity [31,32,33,34]. Many compounds produce use- and frequency-dependent blocks and access to the binding site may occur along different pathways for the neutral and protonated forms of the drug molecule, resulting in different binding affinities, as is the case for lidocaine in interaction with Nav1.5 [35,36,37,38]. Classical studies on the use-dependent block of Na^+^ channels by local anesthetics led to the modulated receptor hypothesis (MRH), claiming that open channel conformations have a higher drug affinity than closed conformations, and inactivation further stabilizes drug binding via the modulation of the binding site configuration, resulting in even higher affinity ([39,40], discussed in [41]). Lateral fenestrations in the permeation domain (S5–S6) of the Nav main subunit facilitate the passage of small neutral drug molecules via a lipophilic pathway to produce a resting state block, although with lower affinity [42]. By contrast, the guarded receptor hypothesis (GRH) postulates that charged compounds can access their binding site only via a hydrophilic pathway, passing through the inner gate during the open state conformation, and subsequently get trapped in the central cavity upon conformational changes to inactivated or closed states [43,44,45,46].

Cenobamate is a novel highly effective third-generation antiseizure drug used for focal onset seizures in adults, particularly in multi-drug-resistant epilepsy [47,48,49]. Chemically it is an alkyl carbamate derivative, featuring a halogenated aromatic ring, a tetrazole heterocycle, and a carbamate group that can be found in several other antiepileptic drugs acting as Na^+^ channels inhibitors, such as carbamazepine and derivatives, carisbamate, felbamate, rufinamide, lacosamide, phenytoin, and others [50]. Its effectiveness is due to actions on multiple targets, including positive allosteric modulatory effects on γ-aminobutyric acid type A (GABA_A_) receptors [51], combined with the inhibition of neuronal Nav channels, particularly of the late or persistent Na^+^ current component, with an apparent IC_50_ of 53.1 ± 4.2 μM in isolated rat hippocampal CA3 neurons [52]. Its pharmacokinetics is complex, with a maximal effective plasma therapeutic concentration of 170 µM for a daily dose of 400 mg, and an average binding to plasma proteins of ~60% [53,54]. Clinical and non-clinical safety studies have evidenced QT interval shortenings with more than 20 ms at doses of 200 and 400 mg/day but not below 300 ms in healthy volunteers [55,56]. These effects led to the prohibition of the drug for patients with hereditary short QT syndrome and careful cardiology monitoring of patients during combined treatment with other antiseizure drugs with similar effects, such as lamotrigine or rufinamide.

Advanced structural methods have provided new insights into the architecture of voltage-dependent Na^+^ channels. The development of X-ray diffraction methods led to a first wave of progress in unraveling the detailed 3D structure of voltage-dependent Na^+^ channels of prokaryotes, with separated 6TM subunits: the NaChBac channel from *Bacillus halodurans* in 2001 [57] and its orthologue NavRh from *Rickettsiales* sp. HIMB114 [58], followed in 2011 by the NavAb channel from *Arcobacter butzleri* RM4018 in a closed conformation ([59] reviewed in [60]) and NavMs from *Magnetococcus* sp. MC-1 [61]. A second wave of progress was fueled by advances in cryoelectron microscopy: a low-resolution structure of voltage-dependent Na^+^ channels from *Electrophorus electricus* [62] was followed by high-resolution structures of NavPaS from *Periplaneta americana* [63], Nav1.4 (α+β) from *Electrophorus electricus* [64] and *Homo sapiens* [65], Nav1.2 [66], Nav1.7 [67,68], Nav1.5 [69,70], Nav1.1 [71], Nav1.8 [72], and Nav1.6 [73]. Together with accurate structure prediction engines such as AlphaFold2 or RoseTTAFold [74] and molecular modeling computational tools, these discoveries have offered us an opportunity to explore the binding and molecular dynamics of cenobamate via in silico approaches using human Nav1.5 atomic resolution structural models. We compared the estimated binding affinities with experimental data we previously obtained and attempted to assess the predicted effects of certain point mutant variants, resulting in changed residues in the vicinity of the putative cenobamate binding site in the central cavity of Nav1.5 channels.

## 2. Results

### 2.1. Nav1.5 Modeling

**Figure 1:**
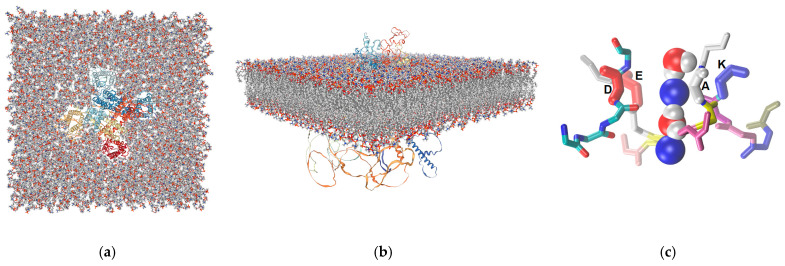
Human Nav1.5 model in open conformation generated by AlphaFold 2 (represented as cartoon and colored in a red to blue colorscale according to the order of amino acids in the sequence) inserted in a DPPC bilayer (represented as licorice with atoms colored according to type): (**a**) top view, showing the channel in the middle of the lipid bilayer; (**b**) side view; (**c**) the file of alternating Na^+^ ions (blue sheres) and water molecules (red and white spheres) placed in the selectivity filter; the residues of the inner charge ring are highlighted and marked.

The human Nav1.5 open-conformation model generated by AlphaFold2 was embedded in an approximately 200 Å × 200 Å square patch of DPPC (Figure 1a,b). An explicit TIP3P water box was added, ensuring a buffer zone of 17.5 Å, and Na^+^ and Cl^−^ ions were added to reach a salt concentration of 0.15 M. In addition, a file of ions was modeled in the selectivity filter of the channel (Figure 1c).

The structural models of eight channels with punctiform mutations were generated after the minimization and equilibration of the wild-type model, as described in the Materials and Methods section. These were further optimized by energy minimization to ensure an easing up of any steric conflicts inflicted by the amino acid side chain changes.

**Figure 2:**
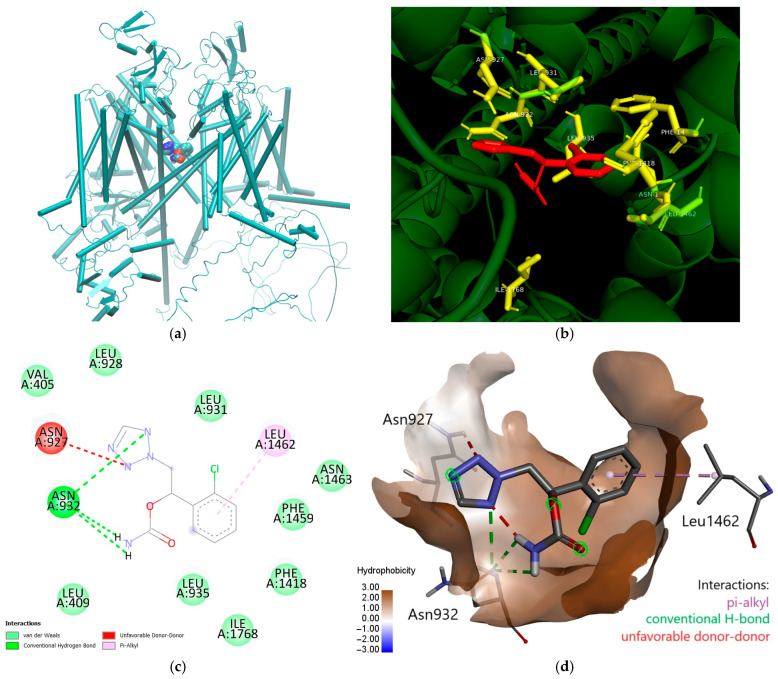
Cenobamate location in the central cavity of energy-optimized and equilibrated Nav1.5 model: (**a**) general view: cenobamate is represented as van der Waals spheres colored according to atom types and the protein is represented as cartoon colored in cyan; (**b**) detailed view of residues located at a distance of less than 4 Å (yellow licorice with labels, while all the protein is represented as green catoon) from the docked cenobamate molecule (red licorice); (**c**) a 2D interaction map between cenobamate and surrounding residues from wild-type Nav1.5 structure in which van der Waals interactions are represented in light green, conventional hydrogen bonds are represented in green, the unfavorable donor-donor interaction is shown in red and the pi-alkyl interaction is shown in pink; (**d**) representation of the surface of cenobamate binding site colored according to the hydrophobicity according to the blue to brown colorscale shown in lower left and cenobamate shown as licorice in the center of the site. Residues Leu1462, Asn932, and Asn927 are represented as licorice, and the interactions established with cenobamate are shown as dashed lines colored according to type (pi-alkyl is pink, conventional H-bonds are green and the unfavorable donor-donor interaction is red).

In the energy-optimized and equilibrated wild-type Nav1.5 model, prior to the production of the 100 ns MD simulation, cenobamate forms two hydrogen bonds with Asn932, a pi–alkyl interaction with Leu1462 and favorable van der Waals interactions with residues Val405, Leu409, Leu928, Leu931, Leu935, Phe1418, Phe1459, Phe1463, and Ile1768. The binding site is mostly hydrophobic (Figure 2). There is a single unfavorable donor–donor interaction with Asn927.

### 2.2. MD Simulations

**Figure 3:**
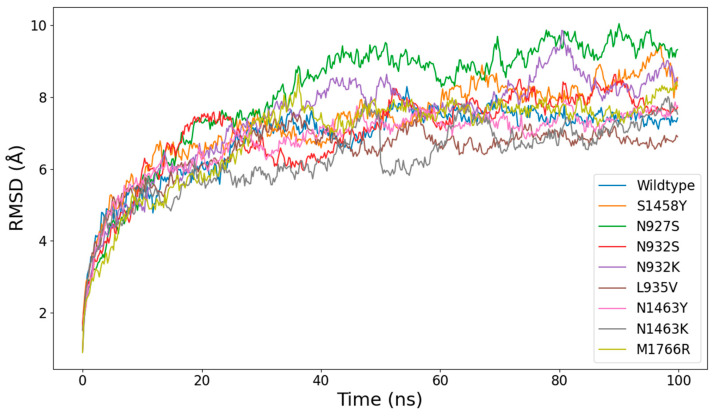
Time series of the RMSD values across a 100 ns simulation, showing the relative stability of the mutant proteins.

**Figure 4:**
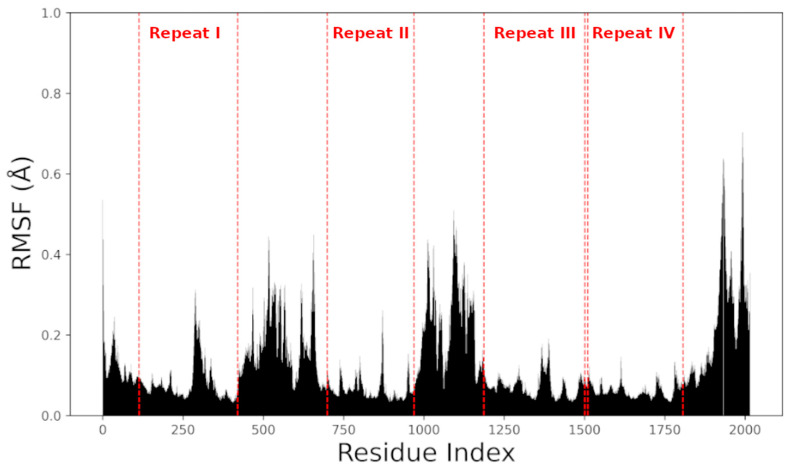
RMSF values of channel protein residues across the 100 ns simulation for the wild-type Nav1.5 model. The transmembrane regions of the four domains (repeats I–IV) are marked and labeled in the figure with red.

All models were further subjected to 100 ns explicit solvent MD simulation runs at constant pressure (1 atm) and temperature (300 K). Over this period, the 9 Nav1.5 models did not suffer major conformational changes, given the size of the unstructured loops on the inner side of the membrane. The RMSDs of each model throughout the simulations are shown in Figure 3. The relatively high RMSD values are due to the flexibility of the above-mentioned stretches and not the transmembrane region. Figure 4 shows that the transmembrane domains (repeats I–IV labeled in the plot) have noticeably lower RMSF values relative to other regions. However, what can be seen is that the N927S and N932K mutations have more influence on the overall flexibility of the system.

**Figure 5:**
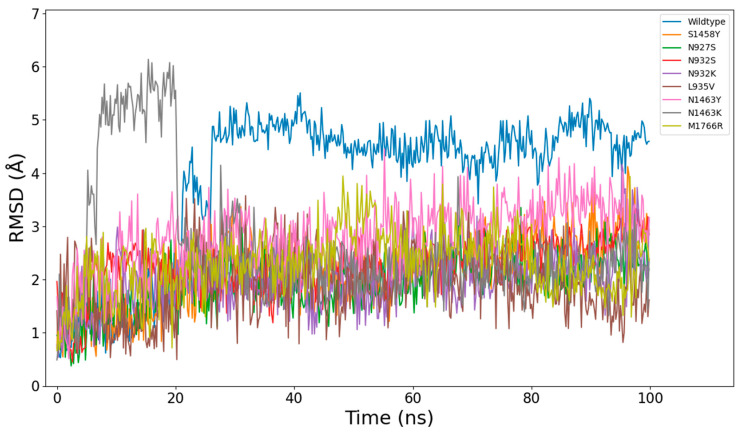
RMSD time series of cenobamate molecules in the systems comprising wild-type and mutant Nav1.5 models calculated over 100 ns, showing how interactions with different receptor residues affect the movements of the ligand.

Focusing on the mutation-induced effects upon the ligand, it is interesting to note that after a longer equilibration of ~20 ns, the flexibility of cenobamate significantly decreases in all mutants when compared to that of wild-type Nav1.5, with RMSDs plotted in blue in Figure 5. Over the equilibration period, only N1463K displays a larger cenobamate flexibility; nevertheless, after that period, it joins the rest of the mutations in constraining the ligand movements. This tighter binding could be an important enthalpic factor explaining the lower estimated *K*_d_ values, as shown below.

### 2.3. Computation of Ligand-Receptor Interaction Energy

The interaction between different Nav1.5 point mutant variants and the cenobamate molecule was estimated by molecular docking assays with a grid box encompassing the central cavity of the channel, which were run in triplicate for each variant, and also via MM-PBSA analysis of MD trajectories. As presented in Table 1, docking assays identified four point mutant variants with cenobamate binding affinity higher than wild-type Nav1.5, while the MM-PBSA results indicate that all mutants had stronger interactions with cenobamate than the wild-type model, with mutant N932S being the top candidate for binding. Contacts between the cenobamate molecule and each of the prepared mutants were computed. The full results are available in Table S1 of Supplementary Data.

### 2.4. Simulations of Action Potential Propagation in a String of Ventricular Cardiomyocytes

**Figure 6:**
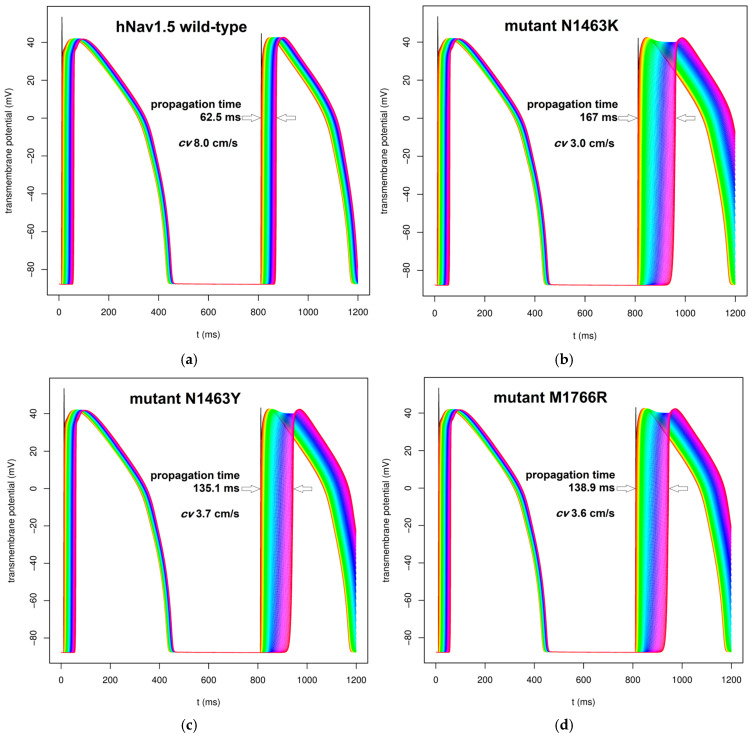
Propagation of AP wavefront along the string of 50 ventricular cardiomyocytes for a cenobamate concentration of 17 µM and an intermyocyte gap junctions coupling conductance *G*_j_ = 1000 pS/pF for different Nav1.5 variants: (**a**) wild-type Nav1.5; (**b**) mutant N1463K (rs1064795922); (**c**) mutant N1463Y (rs199473614); (**d**) mutant M1766R (rs752476527); *cv*—conduction velocity. Each color corresponds to APs generated by a cell in the 50-cell string, in rainbow order. The initial black lines correspond to the moment of current injection. The propagation times are shown in each image and the delays between APs are marked with arrows.

Simulations of action potential (AP) propagation along the linear string of 50 ventricular cardiomyocytes conducted with a modified O’Hara-Rudy 2011 electrophysiology model, using pharmacological data in different conditions of intermyocyte coupling conductance (*G*_j_) for a cenobamate concentration of 17 µM, yielded the results shown in Figure 6. These data indicate differences in propagation delay along the string for high-affinity mutants relative to the models adjusted for wild-type Nav1.5 channels. The computed AP depolarization wavefront conduction velocities (*cv*) over the total string length of 5 mm for the second propagated AP were as follows:

- For *G*_j_ = 1000 pS/pF (a value six-fold lower than normal), the estimated *cv* was 8.0 cm/s for wild-type Nav1.5, 3.0 cm/s for N1463K, 3.7 cm/s for N1463Y, and 3.6 cm/s for M1766R;
- For *G*_j_ = 2000 pS/pF (three-fold lower than normal coupling conductance), the differences between mutants were far less striking: the estimated *cv* was 14.9 cm/s for wild-type Nav1.5, 11.8 cm/s for N1463K, 12.4 cm/s for N1463Y, and 12.4 cm/s for M1766R.

## 3. Discussion

This study was made possible by the recent progress in structural biology, resulting in the availability of accurate 3D data for Nav channels in open conformation [69,70,75], as well as by a variety of effective molecular modeling computational tools. However, highly flexible regions of cryogenic electron microscopy-derived structures, such as the intracellular loops, may be poorly resolved, or some backbone torsion angles may be strained, and these problems may be solved by advanced artificial intelligence-based algorithms such as AlphaFold2. Its predictions are regularly competitive with experimental predictions in CASP14 [76]. We note the recent publication of a molecular dynamics study using a non-conducting Nav1.5 3D model predicted by AlphaFold for long production runs (1 µs) that demonstrated structural stability of the transmembrane region of the channel [77]. Another conclusion of this study was that the AlphaFold prediction of the structure of highly variable cytoplasmic domains was less accurate compared to the more stable transmembrane region. However, for our study, the endodomain of the channel presumably exerts a very limited influence on drug binding within the central cavity. Interestingly, in this MD study, a different four-point three-charge rigid water molecule model (optimal point charge—OPC, [78]) was used, which is considered an optimal match to the ff19SB force field [79]. Again, we do not believe that using the TIP3P water model in our study resulted in significant errors.

For building the lipid bilayer model, we chose dipalmitoyl phosphatidylcholine (DPPC), a phospholipid with saturated fatty acyl chains, generating robust bilayers with less intrinsic fluidity compared to those composed of phospholipids with unsaturated chains such as POPC or POPE. This minimizes the perturbing effects of increased bilayer fluidity or asymmetry induced by unsaturated acyl chains phospholipids, stabilizing the dynamics output. As a long-acyl-chain phospholipid, DPPC also provides a realistic and consistent bilayer thickness [80]. Thus, DPPC has been successfully used in MD simulation studies of ion channels [81,82,83]. It is also worth mentioning that the Nav channel pore is distant from the lipid bilayer; thus, we expect no influence of phospholipid types on cenobamate binding.

It is interesting to analyze the binding site of cenobamate in the Nav1.5 central cavity predicted by molecular docking and further used as the initial position for MD runs (Figure 2c,d). The halogenated aromatic ring is in close vicinity to the side chains of several aromatic residues (Phe1459 and Phe1418 of the S6 helix of domain III), with which it interacts by hydrophobic forces, as well as with other aliphatic hydrophobic side chains (Leu1462 and Leu931). The chloride atom of the ring interacts with the amide group of Asn1463 and with the amino group of Leu935. This explains why the mutation of Asn1463 to tyrosine and particularly to lysine increases the binding affinity, as predicted by docking. The tetrazole heterocycle interacts with other two asparagine residues, Asn927 and Asn932. The secondary group carbonyl of Asn932 also interacts with the nitrogen of the carbamate group of cenobamate, explaining the important effects of substitution mutations of these Asn residues, particularly the high-affinity binding of cenobamate to mutant Asn932Ser predicted by MD simulations. By following MD trajectories, we found that different parts of the ligand molecule, such as the tetrazole ring, as well as their interacting residues, change position over time, while the aromatic ring is more stable. Nevertheless, over the entire duration of simulations, the cenobamate molecule occupies a central position at the inner opening of the selectivity filter in the central cavity, and presumably, it is able to block Na^+^ ions inflow since the sodium ions initially placed in the filter maintain their positions over this time frame. In the above-mentioned MD study of a similar model, it was found that Na^+^ ions did not cross the selectivity filter over a 1 µs time frame [77]. Upon closer inspection of the binding site, it is interesting to note that even though the MM-PBSA calculation indicates stronger binding of all mutants to the cenobamate molecule, the mutated residue may not be directly involved in said stronger binding. For instance, in the case of both N932S and N1463K, the number of frames in which the ligand was bound (within 5 Å) to the mutated residues was similar or lower than to the same residues in the wild-type model. However, in the case of N932K and N1463Y, there was a significantly stronger binding of the mutated residue to the ligand.

Comparing our simulation results with different drug binding sites on Nav channels described in the literature, we conclude that cenobamate occupies the C binding site in the central cavity, more specifically the upper cavity and zones 2 and 3 described by Li et al. [84]. In this study, we did not address the important question of other possible binding sites beyond that in the central cavity, and of possible indirect effects of drug binding on Nav1.5 gating kinetics. It was postulated that local anesthetics and other Nav-inhibiting drugs such as class I antiarrhythmics and some antiseizure compounds [85] interact strongly with some conserved residues of S6 of domain IV (F1760 and Y1767 in the canonical Nav1.5 sequence) as well as L1462 of S6 domain III [32,33,34,36,38,41,43,60,86,87,88] and thus promote fast inactivation, featuring even stronger binding to the inactivated state conformation. According to the MRH, drugs with neutral protonation states that can access the central cavity via a local hydrophobic pathway [40,60] may exert state-dependent binding, while charged compounds follow GRH, quickly entering the cavity during the open state via the internal gate and becoming trapped during inactivated or closed states, thus showing little state dependence of binding affinity [45,87,88]. These differences in blocking mechanism and access pathways result in differential use and frequency dependence, as well as differential binding/unbinding kinetics. Thus, class Ib antiarrhythmics in the Vaughan–Williams classification [89,90,91], such as lidocaine and mexiletine, have the fastest binding kinetics and least use dependency; class Ia drugs such as quinidine and disopyramide are intermediate; and class Ic compounds, like flecainide and encainide, bind and dissociate slowly, exhibiting sodium current inhibition even at normal heart rates; the effects of class Ia and Ic drugs are use-dependent [92]. Our experimental data with cenobamate showing the use-dependent and frequency-dependent inhibition of Nav1.5, together with the electrical neutrality of the drug molecule, indicate at least partially slower access via a lipophilic lateral pathway [93]. However, this molecular modeling study failed to provide evidence of drug interaction with the above-mentioned aromatic residues of S6 of domain IV, the docked position featuring interactions predominantly with S6 of domains II and III, and with a single residue of S6 domain IV: Ile1768. The recent Nav1.5 structures obtained by cryoelectron microscopy provided evidence that the slow activation of S4 voltage sensors of domains III and IV creates a lateral pocket for the binding of the IFMT inactivation particle of the III–IV cytoplasmic loop and decouples the S4-S5 linker of domain III from S6 of domain IV [22,24,70].

We also want to emphasize the very good agreement between the predicted *K*_d_ for the binding of cenobamate to wild-type Nav1.5 channels obtained via molecular docking and the experimental IC_50_ for cenobamate inhibition of Nav1.5 channels obtained in whole-cell patch–clamp experiments [93]: 87.6 µM (experimental) vs. 90.37 µM (molecular docking, Table 1). However, there was less agreement between Gibbs free energies of binding estimates obtained via docking and MD, followed by MM-PBSA for the wild-type Nav1.5 channel model and the eight point mutant variants. Studying the literature, we found large differences between these two methods for other molecular systems as well, e.g., for the interaction of folate receptors α and β with different antifolate compounds [94]. We assume that such differences may result from the approximations in setting relative dielectric constant values for different subdomains of the model when performing continuum electrostatic computations with the MM-PBSA approach [95]. It should also be mentioned that MM-PBSA is a method that provides relative Δ*G*_binding_ values, being useful in classifying different ligand–receptor pairs; for absolute Δ*G*_binding_, one should use more computationally demanding methods such as alchemical absolute binding free energy (ABFE) [96]. Machine learning approaches are also an alternative to improve the accuracy of ligand–receptor interaction energy estimation [97,98], and they have been implemented in a number of accessible computational tools [99,100]. Wang C et al. (2016) analyzed the sources of errors in MM-PBSA computations, identifying a number of factors influencing the overall accuracy of the method, including overlooking contributions to solvation-free energy of buried atoms, approximations introduced by finite difference Poisson–Boltzmann algorithms with various grid spacings, the molecular surface definition based on chosen sets of atomic radii and solvent probe radius (0.6 Å provides satisfactory estimates), the choice of solute dielectric constant (usually *ε*_r_ = 4 for proteins), and particularly the approximation introduced by considering a constant ligand conformational restriction contributing to the protein–ligand interaction free energy based on analysis of a single MD trajectory [95]. Based on methods developed by Gao, Park, and Stern (2010) [101] to estimate the configurational entropy loss due to conformational restriction of the ligand bound to protein compared to its lowest-energy conformation in solution, these authors obtained better estimates of conformational entropy and enthalpy contributions of ligand conformational restraint to Δ*G*_binding_ ([95], reviewed in [102,103]). Other attempts to improve the accuracy of MM-PBSA and MM-GBSA methods, such as polarizable force fields, improved solvation, or quantum-mechanical computations, have failed so far [104]. Although some practical tools and algorithms to improve MM-PBSA free binding energy estimates are available, e.g., [105], it would have been very difficult to adopt them in our study.

## 4. Materials and Methods

We started by selecting several open state conformation structures of Nav1.5 from the RCSB Protein Data Bank (PDB: www.rcsb.org, accessed on 24 November 2024) [106]: PDB ID 7XSU [75] representing rat Nav1.5 (UniProt P15389) fused with a C-terminal rVSV g protein-GFP tag (UniProt B7UCZ6) in interaction with the open-conformation-inducing α-like toxin LQH3 from *Leiurus quinquestriatus hebraeus*, and PDB ID 6UZ0 [70] representing rat Nav1.5 fused with a C-terminal GFP tag from *Aequorea victoria* (UniProt P42212) with flecaininde bound in the central cavity. The corresponding 3D structures of human Nav1.5 in the open state were generated by employing AlphaFold2 [107] and selecting the best structure based on the predicted local distance difference test (pLDDT) score. Mutants were generated using an in-house script with rotamer optimization. The best-ranked AlphaFold2 Nav1.5 protein model was used for incorporation into a DPPC lipid bilayer covering a surface of 200 Å × 200 Å with the Membrane Builder interface of the CHARMM-GUI application (https://charmm-gui.org/, accessed on 24 November 2024) [108,109,110,111,112,113,114,115], following the six consecutive steps: protein PDB input, orienting, establishing system size, building lipid/water components, assembly, and equilibration of the system. Subsequently, we completed the model by adding a file of two sodium ions alternating with water molecules, placed at the origin in the horizontal (XY) plane, parallel to the bilayer, and with the Z positions for ions and oxygens of TIP3P water molecules at mid-distance along the Z axis between the Z position of consecutive planes of 4 backbone carbonyl oxygens of residues within the four domains forming the selectivity filter. After supplementary preprocessing of the structure file containing the Nav1.5 protein, DPPC bilayer, and sodium ions, we used the H++ server available at Virginia Tech (http://newbiophysics.cs.vt.edu/H++/, accessed on 24 November 2024) [116,117,118] to accurately titrate the pKa of ionizable residues using a continuum electrostatic approach based on detailed structural information, computing solvation self-energies and interactions with permanent partial charges as well as with titratable charges [119,120,121]. The output generated by H++ for the titratable protein residues of our model using the linearized Poisson–Boltzmann equations applied to a complete system, including the lipid bilayer with its specific relative dielectric constant instead of the channel protein completely immersed in water, is listed in Table S2 of Supplementary Data; the pK_1/2_ values, compared to the desired pH of 7.2, were used to adjust the protonation state of 7 histidine residues (residues 151, 350, 472, 738, 886, 1200 and 1584) and 2 lysine residues (residues 63 and 1419).

The protein component of the processed model was used for the molecular docking of cenobamate. The cenobamate molecule was converted to the pdbqt format from the 3D conformer downloaded from the PubChem database (https://pubchem.ncbi.nlm.nih.gov, accessed on 24 November 2024) with OpenBabel 3.1.0 [122] and docked on the Nav1.5 model after the addition of polar hydrogens and conversion to the pdbqt format, using MGLTools [123,124]. Molecular docking was performed with AutodockVina 1.2.5 [125,126], using a grid box for placing the ligand in the central cavity of the channel to find the putative drug binding site. Docking was performed in triplicate for all receptor structures, to mediate the stochastic component of the scoring function and provide improved accuracy. By screening several sequence databases such as UniProt [127,128] and ClinVar [129,130], we subsequently identified 8 point mutant human Nav1.5 variants with changed residues in the S6 helices, in the vicinity of the predicted cenobamate binding site, and assessed their effects on cenobamate binding affinity via molecular docking and molecular dynamics approaches.

For MD simulations, the system was prepared using the PACKMOL-Memgen tool [131] included in the AMBERTools23 package [132]. Explicit-type transferable intermolecular potential three-point (TIP3P) water was used, and the membrane bilayer was composed of DPPC lipids. Proteins were parameterized using the ff19SB force field [79] and the lipids were parameterized using the lipid21 force field [133]. The cenobamate ligand was parametrized using GAFF2 [134], with absent parameters generated by homology.

The initial global minimization of the wild-type system was performed using the limited memory Broyden–Fletcher–Goldfarb–Shanno algorithm (L-BFGS) implemented in OpenMM [135]. All the molecular simulations presented in the work were performed using NAMD3 [136]. In order to obtain proper energy distribution of the system, the heating protocol consisted of 4 stages: (1) only the hydrophobic tails of the bilayer were allowed to move; (2) the hydrophobic and hydrophilic parts of the lipid along with the water and ion molecules were allowed to move; (3) the lipids, water, and ions along with the protein side chains and the backbone of the loop regions were allowed to move; and (4) performing the procedure globally, leaving the entire system free. Each of the steps consisted of 1000 steps of conjugate gradient minimization followed by gradual heating to 300K. After the final heating step, a 10 ns equilibration was applied, in order to extract a good starting structure for the MD simulations. All heating/equilibration simulations were run using fully flexible hydrogen bonds, with a 1 fs time step. This was carried out to ensure proper geometry optimization, especially considering the presence of the small ligand.

The Nav1.5 point mutant models were generated using the fully equilibrated wild-type model. Mutant structures were minimized, in order to ensure no errant atomic clashes were present prior to MD simulation. Each of the 9 variants of the Nav1.5 receptor–ligand complexes was subjected to 100 ns of MD simulation with NPT constraints. Considering the size of the system, a time step of 2 fs was used and SHAKE was applied to the hydrogen bonds.

MM-PBSA calculations were performed using the MMPBSA.py program [137]. Since the current work involves a transmembrane channel, the MMPBSA calculation parameters were tuned to reflect the difference in dielectric constant that the presence of the membrane imposes upon the protein’s environment. Parameters were derived from similar use cases in the AMBER user manual as well as other published work [94,95,103], and the script used for computations is provided in Section S1 of Supplementary Data. In total, 50 equally spaced snapshots were used from each simulation.

For the visualization and analysis of molecular structure files, we used VMD (http://www.ks.uiuc.edu/Research/vmd/, accessed on 24 November 2024) [138], PyMol [139], Swiss Model server (https://swissmodel.expasy.org, accessed on 24 November 2024) [140,141,142,143,144,145,146], and BIOVIA Discovery Studio Visualizer v24.1.0.23298, Copyright ©2023, Dessault Systèmes Biovia Corp, free molecular visualization tool (https://discover.3ds.com/discovery-studio-visualizer-download, accessed on 24 November 2024).

In order to assess the effects of increased cenobamate binding affinity for 3 of the tested Nav1.5 point mutants compared to wild-type, as predicted by molecular docking (Table 1), we used a 1D linear ventricular cardiomyocyte string action potential propagation model developed and tested in previous studies [93]. The ventricular tissue model is based on the O’Hara-Rudy 2011 human ventricular cardiomyocyte model [147] with subsequent completions for in silico pharmacology studies [148,149,150]. The string was composed of 50 cardiomyocytes, each with a length of 100 µm, connected by gap junctions with variable conductance ranging from normal values (*G*_j_ = 6000 pS/pF) to 20-fold reduced values specific for ischemic or fibrotic scar myocardial tissue [151,152,153]. The model includes cenobamate blocking and unblocking rates specific for the open and inactivated Nav1.5 channel conformation, estimated from experimental data. To account for increased binding affinity of cenobamate to the three Nav1.5 point mutants relative to wild-type channels we kept constant the blocking rates and modified the unblocking rates to observe the modified *K*_d_ values for these mutants, as shown in Section S2 of Supplementary Data.

## 5. Conclusions

Cenobamate, a novel highly effective antiseizure drug, exerts inhibitory effects on cardiac voltage-dependent Na^+^ channels Nav1.5 at clinically relevant concentrations. Our study using molecular docking and MD approaches for an open-state conformation human Nav1.5 model indicates stable binding of the compound in the central cavity via interactions with multiple aromatic, aliphatic, and secondary amide residues of S6 helices of domains II and III, with the occlusion of the inner outlet of the selectivity filter tract. Some point mutant variants identified in sequence databases UniProt and ClinVar seem to exert even stronger binding of cenobamate than wild-type channels. A computational assessment of the effects of these mutations on AP waveform propagation in a linear string of ventricular cardiomyocytes indicates a significant reduction in conduction velocity for moderately reduced intermyocyte coupling conductances (1000 pS/pF) and low cenobamate concentrations (0.1 × *C*_max_ = 17 µM) that may result in the creation of re-entry loops triggering dangerous ventricular arrhythmias in patients with structural or functional myocardial impairment.

## Figures and Tables

**Table 1:** Results of estimated binding affinity of cenobamate to wild-type hNav1.5 channels and 8 point mutant variants, expressed in binding energy (Kcal/mol) and dissociation constant (*K*_d_), assessed by molecular docking with Autodock Vina 1.2.5 and MD simulations with NAMD, followed by MM-PBSA approaches; mutants with higher binding affinity than wild-type assessed by docking or MM-PBSA are marked with an asterisk.

Mutant	Docking Score (Kcal/mol)	Average Docking Score (Kcal/mol)	Est. *K*_d_ (Docking) (µM)	Δ*G*_binding_ MM-PBSA (Kcal/mol) (Mean ± SD)	Est. *K*_d_ MM-PBSA (µM)
hNav1.5 wild-type	−5.479	−5.50	90.37	−8.8912 ± 2.4548	0.287734
	−5.523				
	−5.489				
N927S (rs199473589)	−5.087	−5.62	73.33 *	−11.6432 ± 2.2684	0.002719 *
	−5.033				
	−6.741				
N932K (rs2125871972)	−5.008	−5.00	208.66	−12.0383 ± 2.3951	0.001392 *
	−4.979				
	−5.022				
N932S (rs2061582195)	−5.096	−5.03	200.57	−14.0262 ± 2.1880	0.000048 *
	−5.080				
	−4.903				
L935V	−4.825	−4.83	279.08	−10.3997 ± 2.8926	0.022348 *
	−4.807				
	−4.862				
S1458Y (rs199473253)	−5.102	−5.11	174.07	−11.8620 ± 2.0938	0.001877 *
	−5.137				
	−5.091				
N1463K (rs1064795922)	−6.110	−6.20	27.62 *	−11.5250 ± 1.9787	0.003322 *
	−6.253				
	−6.227				
N1463Y (rs199473614)	−6.324	−6.01	38.13 *	−9.9455 ± 2.3765	0.048236 *
	−5.536				
	−6.159				
M1766R (rs752476527)	−6.229	−6.03	36.84 *	−9.4284 ± 2.0207	0.115821 *
	−6.068				
	−5.783				

## Data Availability

The original contributions presented in this study are included in the article and Supplementary Materials. Further inquiries can be directed to the corresponding author.

## Supplementary Materials

The following supporting information can be downloaded at: https://www.mdpi.com/article/10.3390/ijms26010358/s1.
