# Supplementary material for: Differential Inhibition by Cenobamate of Canonical Human Nav1.5 Ion Channels and Several Point Mutants
Source: Int J Mol Sci. 2025 Jan 3;26(1):358. doi: 10.3390/ijms26010358 (PMC11720074; doi:10.3390/ijms26010358)
Supplement: Supplementary file 1 [file ijms-26-00358-s001.zip › ijms-3363747-supplementary.pdf]

Supplementary Materials

Differential Inhibition by Cenobamate of Canonical Human Nav1.5 Ion Channels and Several Point Mutants

Table S1 Percentage of contacts between cenobamate and wild-type/mutant Nav1.5 models.

	WT		N927S		N932S		N932K		L935V		S1458Y		N1463Y		N1463K		M1766R	
	Residue	Contacts	Residue	Contacts	Residue	Contacts	Residue	Contacts	Residue	Contacts	Residue	Contacts	Residue	Contacts	Residue	Contacts	Residue	Contacts
Patch 1	ARG367	0.00%	ARG367	0.00%	ARG367	0.00%	ARG367	0.00%	ARG367	0.00%	ARG367	0.00%	ARG367	0.00%	ARG367	1.90%	ARG367	0.00%
	LEU368	0.20%	LEU368	0.00%	LEU368	2.40%	LEU368	0.00%	LEU368	0.00%	LEU368	0.00%	LEU368	0.10%	LEU368	35.40%	LEU368	2.10%
	MET369	19.00%	MET369	72.40%	MET369	89.60%	MET369	15.80%	MET369	60.20%	MET369	68.00%	MET369	26.10%	MET369	99.40%	MET369	65.00%
	THR370	24.70%	THR370	100.00%	THR370	99.40%	THR370	100.00%	THR370	100.00%	THR370	100.00%	THR370	100.00%	THR370	99.90%	THR370	97.80%
	GLN371	49.10%	GLN371	100.00%	GLN371	100.00%	GLN371	100.00%	GLN371	100.00%	GLN371	100.00%	GLN371	100.00%	GLN371	100.00%	GLN371	100.00%
	ASP372	7.80%	ASP372	14.40%	ASP372	19.20%	ASP372	68.60%	ASP372	0.20%	ASP372	13.60%	ASP372	1.60%	ASP372	4.20%	ASP372	9.20%
	TRP374	0.00%	TRP374	0.00%	TRP374	0.00%	TRP374	0.00%	TRP374	0.00%	TRP374	0.00%	TRP374	0.00%	TRP374	12.00%	TRP374	0.00%
	VAL396	0.00%	VAL396	0.00%	VAL396	0.00%	VAL396	0.00%	VAL396	0.00%	VAL396	0.00%	VAL396	0.00%	VAL396	0.10%	VAL396	0.00%
	ILE397	0.60%	ILE397	1.00%	ILE397	0.10%	ILE397	0.20%	ILE397	0.20%	ILE397	0.30%	ILE397	0.00%	ILE397	13.20%	ILE397	2.70%
	SER401	30.10%	SER401	88.60%	SER401	67.30%	SER401	74.50%	SER401	65.70%	SER401	71.00%	SER401	37.30%	SER401	67.60%	SER401	60.90%
	PHE402	98.30%	PHE402	99.90%	PHE402	100.00%	PHE402	98.90%	PHE402	95.00%	PHE402	99.50%	PHE402	96.60%	PHE402	100.00%	PHE402	99.30%
	TYR403	0.10%	TYR403	0.00%	TYR403	1.90%	TYR403	0.00%	TYR403	0.00%	TYR403	0.00%	TYR403	0.00%	TYR403	0.20%	TYR403	0.30%
	LEU404	0.10%	LEU404	0.00%	LEU404	0.00%	LEU404	0.00%	LEU404	0.00%	LEU404	0.00%	LEU404	0.00%	LEU404	0.00%	LEU404	0.00%
	VAL405	100.00%	VAL405	100.00%	VAL405	100.00%	VAL405	100.00%	VAL405	100.00%	VAL405	100.00%	VAL405	37.30%	VAL405	100.00%	VAL405	100.00%
	ASN406	63.70%	ASN406	100.00%	ASN406	100.00%	ASN406	100.00%	ASN406	100.00%	ASN406	100.00%	ASN406	96.60%	ASN406	94.10%	ASN406	99.90%
	ILE408	1.60%	ILE408	2.20%	ILE408	0.10%	ILE408	0.10%	ILE408	0.60%	ILE408	0.30%	ILE408	0.20%	ILE408	0.00%	ILE408	0.50%
	LEU409	100.00%	LEU409	99.80%	LEU409	99.90%	LEU409	33.60%	LEU409	94.10%	LEU409	99.60%	LEU409	99.90%	LEU409	77.50%	LEU409	94.30%
Patch 2	ILE894	0.00%	ILE894	0.00%	ILE894	5.00%	ILE894	0.00%	ILE894	0.00%	ILE894	0.00%	ILE894	0.00%	ILE894	0.80%	ILE894	0.00%
	LEU895	9.20%	LEU895	2.50%	LEU895	20.10%	LEU895	0.00%	LEU895	0.40%	LEU895	0.00%	LEU895	0.20%	LEU895	9.20%	LEU895	0.90%
	CYS896	43.70%	CYS896	100.00%	CYS896	96.50%	CYS896	100.00%	CYS896	99.90%	CYS896	100.00%	CYS896	96.00%	CYS896	51.00%	CYS896	98.10%
	GLY897	59.20%	GLY897	100.00%	GLY897	100.00%	GLY897	100.00%	GLY897	100.00%	GLY897	99.90%	GLY897	99.70%	GLY897	86.30%	GLY897	100.00%
	GLU898	34.30%	GLU898	98.10%	GLU898	29.60%	GLU898	99.60%	GLU898	98.80%	GLU898	99.70%	GLU898	74.50%	GLU898	10.70%	GLU898	97.00%
	TRP899	0.90%	TRP899	10.70%	TRP899	6.80%	TRP899	20.70%	TRP899	1.20%	TRP899	12.60%	TRP899	0.40%	TRP899	4.60%	TRP899	15.70%
	MET923	18.00%	MET923	91.80%	MET923	91.60%	MET923	82.40%	MET923	99.60%	MET923	58.00%	MET923	98.70%	MET923	80.60%	MET923	91.80%
	VAL924	0.00%	VAL924	0.00%	VAL924	0.00%	VAL924	0.00%	VAL924	0.00%	VAL924	0.00%	VAL924	0.00%	VAL924	0.00%	VAL924	0.40%
	ASN927	92.90%	**SER927**	63.20%	ASN927	97.00%	ASN927	100.00%	ASN927	100.00%	ASN927	100.00%	ASN927	100.00%	ASN927	65.90%	ASN927	99.50%
	LEU928	65.40%	LEU928	99.60%	LEU928	99.80%	LEU928	99.10%	LEU928	100.00%	LEU928	98.00%	LEU928	99.60%	LEU928	80.70%	LEU928	99.90%
	LEU931	43.50%	LEU931	63.20%	LEU931	99.80%	LEU931	89.50%	LEU931	41.00%	LEU931	98.20%	LEU931	1.80%	LEU931	1.10%	LEU931	2.80%
	ASN932	14.30%	ASN932	99.60%	**SER932**	0.00%	**LYS932**	99.90%	ASN932	24.20%	ASN932	30.60%	ASN932	3.60%	ASN932	12.20%	ASN932	14.20%
	LEU935	32.70%	LEU935	10.60%	LEU935	0.00%	LEU935	18.90%	**VAL935**	18.00%	LEU935	9.70%	LEU935	29.20%	LEU935	0.00%	LEU935	0.50%
Patch 3	TRP1345	0.00%	TRP1345	0.00%	TRP1345	0.00%	TRP1345	0.00%	TRP1345	0.00%	TRP1345	0.00%	TRP1345	0.30%	TRP1345	0.10%	TRP1345	0.00%
	VAL1415	2.70%	VAL1415	9.20%	VAL1415	19.30%	VAL1415	6.60%	VAL1415	12.10%	VAL1415	17.40%	VAL1415	16.80%	VAL1415	5.60%	VAL1415	12.10%
	ALA1416	95.70%	ALA1416	100.00%	ALA1416	99.40%	ALA1416	99.10%	ALA1416	99.90%	ALA1416	100.00%	ALA1416	100.00%	ALA1416	87.20%	ALA1416	89.80%
	THR1417	96.40%	THR1417	100.00%	THR1417	100.00%	THR1417	100.00%	THR1417	100.00%	THR1417	100.00%	THR1417	100.00%	THR1417	99.90%	THR1417	100.00%
	PHE1418	99.90%	PHE1418	100.00%	PHE1418	100.00%	PHE1418	100.00%	PHE1418	100.00%	PHE1418	99.80%	PHE1418	100.00%	PHE1418	100.00%	PHE1418	99.70%
	LYS1419	12.20%	LYS1419	59.10%	LYS1419	15.30%	LYS1419	29.70%	LYS1419	40.60%	LYS1419	53.20%	LYS1419	37.10%	LYS1419	4.10%	LYS1419	53.10%
	ILE1454	0.10%	ILE1454	0.00%	ILE1454	0.20%	ILE1454	0.00%	ILE1454	0.00%	ILE1454	0.00%	ILE1454	0.00%	ILE1454	0.00%	ILE1454	0.00%
	SER1458	18.90%	SER1458	7.00%	SER1458	69.40%	SER1458	5.60%	SER1458	21.40%	**TYR1458**	99.90%	SER1458	74.90%	SER1458	1.70%	SER1458	33.40%
	PHE1459	93.10%	PHE1459	72.50%	PHE1459	12.30%	PHE1459	1.50%	PHE1459	71.40%	PHE1459	0.00%	PHE1459	92.80%	PHE1459	86.40%	PHE1459	62.30%
	PHE1460	0.10%	PHE1460	0.00%	PHE1460	0.00%	PHE1460	0.00%	PHE1460	0.00%	PHE1460	0.00%	PHE1460	0.00%	PHE1460	0.00%	PHE1460	0.00%
	LEU1462	98.80%	LEU1462	99.90%	LEU1462	99.40%	LEU1462	99.70%	LEU1462	98.80%	LEU1462	100.00%	LEU1462	99.90%	LEU1462	84.60%	LEU1462	83.50%
	ASN1463	48.50%	ASN1463	0.50%	ASN1463	5.80%	ASN1463	5.90%	ASN1463	7.10%	ASN1463	7.20%	**TYR1463**	93.90%	**LYS1463**	**19.30%**	LYS1463	68.30%
	PHE1465	0.60%	PHE1465	0.00%	PHE1465	0.00%	PHE1465	0.00%	PHE1465	0.00%	PHE1465	0.00%	PHE1465	0.00%	PHE1465	0.00%	PHE1465	0.00%
	ILE1466	92.90%	ILE1466	81.60%	ILE1466	90.70%	ILE1466	95.80%	ILE1466	85.40%	ILE1466	97.70%	ILE1466	94.20%	ILE1466	64.60%	ILE1466	86.40%

Patch 4	ILE1707	30.10%	ILE1707	0.00%	ILE1707	0.00%	ILE1707	0.00%	ILE1707	0.00%	ILE1707	0.00%	ILE1707	0.00%	ILE1707	8.30%	ILE1707	0.00%
	THR1708	54.30%	THR1708	0.40%	THR1708	1.50%	THR1708	0.00%	THR1708	0.00%	THR1708	0.10%	THR1708	0.30%	THR1708	20.50%	THR170	7.30%
	THR1709	90.10%	THR1709	86.00%	THR1709	74.70%	THR1709	14.70%	THR1709	39.00%	THR1709	89.80%	THR1709	79.40%	THR1709	68.70%	THR170	67.80%
	SER1710	99.10%	SER1710	100.00%	SER1710	92.00%	SER1710	32.90%	SER1710	71.00%	SER1710	100.00%	SER1710	100.00%	SER1710	100.00%	SER171	86.20%
	ALA1711	0.40%	ALA1711	0.00%	ALA1711	4.00%	ALA1711	0.00%	ALA1711	0.20%	ALA1711	0.10%	ALA1711	0.40%	ALA1711	9.70%	ALA171	0.10%
	TRP1713	0.00%	TRP1713	0.00%	TRP1713	0.00%	TRP1713	0.00%	TRP1713	0.00%	TRP1713	0.00%	TRP1713	0.00%	TRP1713	0.20%	TRP171	0.00%
	ILE1756	29.90%	ILE1756	0.10%	ILE1756	0.00%	ILE1756	0.00%	ILE1756	0.00%	ILE1756	0.00%	ILE1756	0.00%	ILE1756	19.40%	ILE1756	3.90%
	SER1759	4.30%	SER1759	0.00%	SER1759	0.00%	SER1759	0.00%	SER1759	0.00%	SER1759	0.00%	SER1759	0.00%	SER1759	13.50%	SER175	0.10%
	PHE1760	100.00%	PHE1760	100.00%	PHE1760	100.00%	PHE1760	100.00%	PHE1760	100.00%	PHE1760	100.00%	PHE1760	99.90%	PHE1760	99.90%	PHE176	99.60%
	VAL1763	78.00%	VAL1763	70.10%	VAL1763	79.60%	VAL1763	40.80%	VAL1763	52.70%	VAL1763	42.30%	VAL1763	38.90%	VAL1763	37.40%	VAL176	35.60%
	VAL1764	100.00%	VAL1764	100.00%	VAL1764	100.00%	VAL1764	100.00%	VAL1764	100.00%	VAL1764	100.00%	VAL1764	100.00%	VAL1764	99.90%	VAL176	100.00%
	ASN1765	0.60%	ASN1765	0.00%	ASN1765	0.00%	ASN1765	0.00%	ASN1765	0.00%	ASN1765	0.00%	ASN1765	0.00%	ASN1765	0.00%	ASN176	0.00%
	TYR1767	0.30%	TYR1767	0.10%	TYR1767	0.50%	TYR1767	0.00%	TYR1767	0.00%	TYR1767	0.00%	TYR1767	0.00%	TYR1767	0.00%	TYR176	0.00%
	ILE1768	74.30%	ILE1768	94.90%	ILE1768	95.60%	ILE1768	99.80%	ILE1768	99.80%	ILE1768	99.50%	ILE1768	96.60%	ILE1768	73.30%	ILE1768	90.60%

Table S2. Protonation state of hNav1.5 residues computed with H++ for the channel embedded in a DPPC lipid bilayer. Histidine and lysine residues with pK1/2 values exceeding the pH set to 7.2 are marked in red, and their protonation state were corrected accordingly.

Residue		pK_{int}	$pK_{1/2}$	Residue		pK_{int}	$pK_{1/2}$	Residue		pK_{int}	$pK_{1/2}$
ARG	8	11.694	>12	ASP	1690	4.747	1.471	GLU	1867	4.987	5.472
ARG	14	12.163	>12	ASP	1714	2.306	<0	GLU	1876	5.074	4.44
ARG	15	12.206	>12	ASP	1729	3.919	3.092	GLU	1877	4.553	4.089
ARG	18	11.979	>12	ASP	1741	5.348	5.497	GLU	1890	4.537	4.203
ARG	27	11.860	>12	ASP	1789	4.804	<0	GLU	1901	4.856	3.616
ARG	34	12.010	>12	ASP	1790	4.807	5.161	GLU	1902	4.738	2.851
ARG	43	12.080	>12	ASP	1792	4.713	3.12	GLU	1938	4.325	4.469
ARG	53	11.958	>12	ASP	1802	2.456	0.556	GLU	1939	4.514	4.436
ARG	104	10.799	>12	ASP	1816	4.671	2.913	GLU	1943	5.083	1.98
ARG	121	13.826	>12	ASP	1819	4.619	2.477	GLU	1945	4.203	3.987
ARG	122	12.908	>12	ASP	1839	4.082	2.912	GLU	1954	5.147	4.696
ARG	179	11.992	>12	ASP	1846	4.140	2.713	GLU	1999	4.296	4.341
ARG	190	11.416	>12	ASP	1852	5.339	1.28	GLU	2013	4.490	4.468
ARG	219	12.522	>12	ASP	1869	4.259	3.021	HID	118	3.276	<0
ARG	222	12.934	>12	ASP	1940	4.063	3.437	HID	130	6.136	1.668
ARG	225	11.657	>12	ASP	1978	3.910	3.632	HID	151	5.994	8.289
ARG	277	11.352	>12	ASP	1986	3.748	3.685	HID	184	5.212	2.832
ARG	282	12.170	>12	ASP	1994	4.013	3.93	HID	278	7.355	6.711
ARG	340	12.049	>12	ASP	2000	3.723	3.668	HID	350	5.564	9.42
ARG	367	9.064	>12	ASP	2003	3.984	4.028	HID	445	6.618	6.594
ARG	376	10.452	>12	ASP	2009	3.932	3.647	HID	472	7.023	7.203
ARG	383	12.196	>12	ASP	2011	3.861	3.109	HID	508	6.533	6.478
ARG	433	11.312	>12	CYS	139	12.398	>12	HID	557	7.047	6.925
ARG	451	11.834	>12	CYS	145	11.770	>12	HID	558	7.164	6.935
ARG	458	12.048	>12	CYS	182	9.864	8.496	HID	585	6.755	6.798
ARG	474	11.949	>12	CYS	260	11.101	>12	HID	588	6.737	6.164
ARG	475	11.904	11.956	CYS	373	8.044	>12	HID	617	6.891	6.768
ARG	478	12.262	11.778	CYS	489	8.203	8.813	HID	626	6.849	6.18
ARG	479	12.312	>12	CYS	597	8.506	9.192	HID	681	6.577	6.894
ARG	481	11.880	>12	CYS	649	8.098	8.375	HID	738	6.906	8.266
ARG	493	11.861	>12	CYS	683	9.427	9.41	HID	880	5.521	5.558
ARG	504	11.898	>12	CYS	686	12.780	>12	HID	886	5.004	8.666
ARG	513	11.655	11.895	CYS	699	9.143	11.416	HID	1200	7.400	7.233
ARG	517	11.805	11.896	CYS	700	7.067	7.303	HID	1204	6.981	6.825
ARG	523	11.461	11.533	CYS	726	10.714	>12	HID	1584	8.988	8.429
ARG	526	11.844	11.97	CYS	896	10.879	>12	HID	1849	4.498	6.726
ARG	533	11.936	11.784	CYS	981	10.041	10.096	HID	1900	6.816	7.141
ARG	534	11.941	11.952	CYS	982	11.080	>12	HID	1915	7.004	6.255
ARG	535	11.823	>12	CYS	1004	8.482	8.47	HID	1923	6.087	5.941
ARG	568	11.871	11.777	CYS	1046	8.828	9.015	HID	1997	6.578	6.993
ARG	569	11.972	11.905	CYS	1128	8.248	8.51	LYS	26	10.293	10.593
ARG	620	12.023	>12	CYS	1136	8.560	8.962	LYS	31	9.538	9.994
ARG	659	11.876	11.945	CYS	1167	11.349	>12	LYS	62	10.385	10.314
ARG	661	12.450	>12	CYS	1172	7.756	7.21	LYS	63	7.961	6.891
ARG	680	12.726	>12	CYS	1176	12.163	>12	LYS	91	9.091	9.585
ARG	689	12.210	>12	CYS	1178	13.200	>12	LYS	98	9.250	8.409
ARG	693	10.672	11.772	CYS	1179	9.944	>12	LYS	100	10.385	11.751
ARG	800	10.833	11.058	CYS	1198	14.215	>12	LYS	126	12.180	>12
ARG	808	12.353	>12	CYS	1272	12.154	>12	LYS	158	10.573	11.635

ARG	811	12.487	>12	CYS	1341	11.056	>12	LYS	175	10.481	>12
ARG	814	12.586	>12	CYS	1539	11.530	>12	LYS	228	9.859	>12
ARG	869	12.236	>12	CYS	1575	13.009	>12	LYS	237	10.444	10.874
ARG	878	9.764	>12	CYS	1703	12.588	>12	LYS	248	9.351	9.97
ARG	893	9.057	>12	CYS	1850	10.807	>12	LYS	249	10.391	>12
ARG	954	11.869	>12	GLU	19	4.332	3.634	LYS	279	10.856	>12
ARG	965	10.285	11.012	GLU	25	4.624	2.261	LYS	317	9.431	>12
ARG	968	11.727	>12	GLU	30	4.832	3.422	LYS	343	10.155	>12
ARG	971	12.993	>12	GLU	41	4.665	4.62	LYS	387	12.090	>12
ARG	975	11.508	>12	GLU	44	4.526	4.327	LYS	430	9.747	>12
ARG	986	12.369	>12	GLU	48	4.400	3.905	LYS	432	10.194	11.786
ARG	988	11.975	>12	GLU	49	4.404	3.945	LYS	442	10.317	11.25
ARG	1023	11.799	>12	GLU	50	4.481	3.676	LYS	443	10.268	11.358
ARG	1027	11.845	>12	GLU	74	4.669	3.344	LYS	477	10.231	9.84
ARG	1093	12.282	>12	GLU	78	7.460	3.329	LYS	480	10.171	9.522
ARG	1116	11.937	>12	GLU	81	4.919	3.542	LYS	496	10.348	10.444
ARG	1174	11.668	>12	GLU	161	6.221	1.173	LYS	521	10.197	10.056
ARG	1175	13.149	>12	GLU	171	8.039	<0	LYS	590	10.237	9.668
ARG	1193	10.822	11.026	GLU	208	6.299	3.555	LYS	591	10.234	10.193
ARG	1195	13.154	>12	GLU	295	4.602	4.737	LYS	682	9.103	9.686
ARG	1232	11.559	>12	GLU	302	4.414	4.561	LYS	707	10.057	>12
ARG	1303	12.915	>12	GLU	312	4.518	4.773	LYS	711	10.396	>12
ARG	1306	12.569	>12	GLU	337	4.448	4.265	LYS	767	10.093	>12
ARG	1309	11.604	>12	GLU	346	4.648	3.905	LYS	817	9.501	>12
ARG	1312	13.539	>12	GLU	375	2.263	<0	LYS	820	9.922	11.487
ARG	1316	12.235	>12	GLU	417	4.651	5.126	LYS	830	9.946	>12
ARG	1321	11.919	>12	GLU	418	5.406	2.709	LYS	863	10.502	11.083
ARG	1362	11.211	>12	GLU	426	5.578	1.573	LYS	974	9.813	>12
ARG	1432	14.467	>12	GLU	428	4.810	5.004	LYS	991	10.266	10.364
ARG	1512	12.406	>12	GLU	429	4.769	3.638	LYS	1018	10.160	10.59
ARG	1583	11.121	11.515	GLU	431	4.678	3.756	LYS	1024	10.308	9.948
ARG	1623	12.767	>12	GLU	436	4.723	4.14	LYS	1075	10.433	10.735
ARG	1626	13.326	>12	GLU	439	4.601	3.816	LYS	1120	10.185	10.403
ARG	1629	13.595	>12	GLU	444	5.546	1.966	LYS	1162	10.500	>12
ARG	1632	11.889	>12	GLU	446	4.882	3.806	LYS	1189	9.961	9.432
ARG	1635	11.588	>12	GLU	462	4.461	4.339	LYS	1196	10.108	10.652
ARG	1638	11.763	>12	GLU	473	4.358	3.294	LYS	1233	10.296	10.539
ARG	1644	12.143	>12	GLU	487	5.679	5.9	LYS	1236	10.419	>12
ARG	1739	12.030	>12	GLU	488	3.647	3.569	LYS	1244	11.109	>12
ARG	1826	12.085	>12	GLU	491	4.397	4.844	LYS	1257	11.681	>12
ARG	1847	11.968	>12	GLU	500	4.326	4.852	LYS	1264	10.426	>12
ARG	1860	11.721	>12	GLU	540	4.252	4.517	LYS	1265	10.478	11.573
ARG	1897	11.749	>12	GLU	547	4.339	4.683	LYS	1300	10.649	>12
ARG	1898	11.193	>12	GLU	553	4.312	4.232	LYS	1359	11.187	>12
ARG	1910	12.188	11.784	GLU	555	4.204	4.015	LYS	1381	9.634	11.847
ARG	1913	11.880	11.987	GLU	610	4.392	4.339	LYS	1397	9.959	>12
ARG	1914	12.270	11.942	GLU	625	4.678	4.229	LYS	1399	9.910	>12
ARG	1919	11.736	>12	GLU	635	4.397	4.482	LYS	1419	1.691	7.213
ARG	1929	12.047	>12	GLU	636	4.223	4.295	LYS	1477	10.370	>12
ARG	1944	11.709	>12	GLU	654	4.258	4.228	LYS	1478	10.328	>12
ARG	1958	12.356	>12	GLU	655	4.298	4.016	LYS	1479	10.281	11.492
ARG	1982	12.000	>12	GLU	674	4.643	4.968	LYS	1492	10.348	>12
ARG	1991	11.909	>12	GLU	675	4.284	3.38	LYS	1493	9.423	>12
ARG	2010	11.912	>12	GLU	677	4.855	4.257	LYS	1499	10.278	9.828
ARG	2012	11.968	>12	GLU	678	4.720	4.296	LYS	1500	9.984	>12

ASP	57	4.517	0.348	GLU	698	4.364	3.711	LYS	1504	9.590	>12
ASP	66	4.333	1.362	GLU	737	6.723	5.033	LYS	1505	10.288	10.934
ASP	82	5.523	0.7	GLU	744	3.872	3.436	LYS	1508	10.437	11.394
ASP	84	4.499	<0	GLU	746	4.730	3.601	LYS	1516	8.973	8.653
ASP	152	3.962	1.452	GLU	747	4.906	5.318	LYS	1527	11.863	>12
ASP	191	4.794	<0	GLU	763	8.240	<0	LYS	1556	9.195	10.568
ASP	197	6.955	<0	GLU	795	8.260	1.159	LYS	1562	11.298	11.722
ASP	211	4.968	4.047	GLU	867	4.850	4.302	LYS	1578	10.158	>12
ASP	252	5.921	4.01	GLU	898	4.267	2.99	LYS	1614	9.741	10.263
ASP	297	4.009	3.649	GLU	901	3.090	<0	LYS	1641	10.539	>12
ASP	305	3.899	3.474	GLU	908	4.792	5.484	LYS	1683	10.191	>12
ASP	310	4.496	4.96	GLU	952	4.198	4.583	LYS	1800	10.216	11.636
ASP	322	4.239	<0	GLU	955	4.721	3.665	LYS	1829	9.955	>12
ASP	331	5.859	4.379	GLU	1015	4.457	4.434	LYS	1859	9.688	>12
ASP	349	4.920	<0	GLU	1017	4.514	4.177	LYS	1872	9.797	>12
ASP	356	3.815	<0	GLU	1025	4.320	3.726	LYS	1878	9.937	9.984
ASP	372	2.123	<0	GLU	1029	4.645	4.675	LYS	1886	10.286	11.1
ASP	454	4.639	4.132	GLU	1030	4.266	4.247	LYS	1899	9.319	>12
ASP	492	3.827	3.372	GLU	1032	4.480	4.475	LYS	1922	10.413	10.29
ASP	498	3.769	3.654	GLU	1043	4.449	4.57	TYR	68	11.462	>12
ASP	501	3.891	3.92	GLU	1053	4.356	4.488	TYR	87	10.787	10.84
ASP	536	4.162	3.542	GLU	1060	4.326	5.138	TYR	112	13.279	>12
ASP	542	3.693	3.752	GLU	1061	4.022	3.556	TYR	159	10.861	10.971
ASP	545	3.778	4.139	GLU	1063	3.281	0.982	TYR	162	12.124	>12
ASP	546	3.729	4.117	GLU	1064	5.816	2.177	TYR	168	13.277	>12
ASP	596	4.155	3.071	GLU	1070	4.516	4.333	TYR	205	12.607	>12
ASP	608	3.889	3.336	GLU	1071	4.404	4.504	TYR	307	14.182	>12
ASP	629	4.331	2.791	GLU	1072	4.294	4.307	TYR	314	15.489	>12
ASP	651	3.823	3.79	GLU	1078	4.107	4.048	TYR	339	10.822	10.587
ASP	716	5.381	4.458	GLU	1087	4.295	4.142	TYR	352	11.796	>12
ASP	720	4.915	0.103	GLU	1105	4.277	4.322	TYR	378	14.315	>12
ASP	772	5.741	2.192	GLU	1107	4.348	4.432	TYR	389	15.133	>12
ASP	785	8.363	2.653	GLU	1122	4.327	4.337	TYR	403	13.708	>12
ASP	870	3.753	3.324	GLU	1130	4.322	4.221	TYR	416	11.399	>12
ASP	872	3.829	3.964	GLU	1133	4.376	4.856	TYR	694	13.696	>12
ASP	883	4.329	<0	GLU	1138	4.403	4.445	TYR	739	10.251	>12
ASP	905	3.596	1.415	GLU	1149	4.077	4.103	TYR	774	10.173	>12
ASP	945	4.802	4.612	GLU	1152	4.340	4.552	TYR	775	10.089	11.777
ASP	951	3.540	2.575	GLU	1165	5.293	3.392	TYR	776	14.759	>12
ASP	953	3.868	2.729	GLU	1170	4.192	3.321	TYR	865	11.713	>12
ASP	979	4.807	3.633	GLU	1203	5.701	1.778	TYR	1009	9.483	9.388
ASP	1041	4.056	4.02	GLU	1208	6.478	0.26	TYR	1199	12.836	>12
ASP	1055	3.969	4.064	GLU	1225	7.088	6.453	TYR	1228	10.732	11.994
ASP	1057	3.928	4.222	GLU	1230	6.217	5.479	TYR	1241	12.626	>12
ASP	1058	4.017	4.207	GLU	1231	4.480	3.607	TYR	1248	15.063	>12
ASP	1062	4.205	4.804	GLU	1240	5.996	2.919	TYR	1261	12.123	>12
ASP	1091	4.567	4.396	GLU	1253	9.270	<0	TYR	1266	15.023	>12
ASP	1114	3.754	3.514	GLU	1295	4.953	3.641	TYR	1375	10.460	10.871
ASP	1134	3.879	3.963	GLU	1318	5.089	3.677	TYR	1394	10.703	11.55
ASP	1143	3.926	3.919	GLU	1368	4.546	4.784	TYR	1409	15.410	>12
ASP	1156	3.865	3.626	GLU	1385	4.729	4.005	TYR	1426	9.227	>12
ASP	1160	4.123	3.467	GLU	1392	4.699	4.835	TYR	1434	11.201	>12
ASP	1163	4.544	2.993	GLU	1435	4.534	3.325	TYR	1442	10.153	10.375
ASP	1166	4.151	2.415	GLU	1436	5.418	4.324	TYR	1445	11.635	>12
ASP	1182	3.960	3.607	GLU	1441	6.475	1.747	TYR	1447	12.591	>12

ASP	1226	4.563	1.34	GLU	1489	4.702	3.794	TYR	1449	14.491	>12
ASP	1243	7.375	<0	GLU	1490	4.430	4.562	TYR	1494	11.489	>12
ASP	1275	8.024	0.944	GLU	1548	6.871	2.582	TYR	1495	11.285	>12
ASP	1280	7.922	6.97	GLU	1555	4.391	3.632	TYR	1517	13.061	>12
ASP	1370	4.303	2.672	GLU	1574	7.678	2.278	TYR	1585	10.433	10.089
ASP	1403	4.348	0.223	GLU	1685	5.479	4.312	TYR	1586	16.384	>12
ASP	1423	3.112	<0	GLU	1773	6.488	0.858	TYR	1615	10.139	9.876
ASP	1430	5.492	<0	GLU	1780	5.205	6.584	TYR	1671	12.538	>12
ASP	1471	4.764	3.836	GLU	1781	5.691	4.256	TYR	1681	11.907	>12
ASP	1484	4.577	1.89	GLU	1784	5.424	6.936	TYR	1727	10.862	>12
ASP	1523	6.084	3.643	GLU	1788	4.987	<0	TYR	1755	16.036	>12
ASP	1531	5.459	0.69	GLU	1796	4.811	3.471	TYR	1767	13.691	>12
ASP	1550	5.203	<0	GLU	1799	5.555	5.884	TYR	1795	14.113	>12
ASP	1551	3.889	1.851	GLU	1804	4.267	4.254	TYR	1811	10.283	10.605
ASP	1595	8.540	<0	GLU	1810	4.106	4.266	TYR	1889	11.193	>12
ASP	1610	6.616	4.391	GLU	1823	4.427	3.508	TYR	1950	9.803	10.153
ASP	1689	4.727	1.011	GLU	1864	4.684	3.315	TYR	1977	9.844	10.192
								TYR	1995	9.534	9.821

Section S1. Input file for MM-GB(PB)SA calculation

```
&general
  endframe=500, verbose=1,
  interval=10,
  strip_mask=":WAT:PA:PC:Na+:Cl-"
/

&gb
 igb=5, saltcon=0.150
/

&pb
 istrng=0.150, radiopt=0,
 indi=20.0, fillratio=1.25, memopt=1,
 ipb=1, bcopt=10, solvopt=2, nfocus=1,
 poretype=1, mctrdz=132,
 mthick=40, emem=35.0, cutnb=0,
 eneopt=2, maxarcdot=15000
/
```

Section S2. Computation of state-specific cenobamate blocking and unblocking rates for the open and inactivated Nav1.5 channel conformation of wild-type channels and point mutant variants N1463K, N1463Y, and M1766R

We began with the state-specific cenobamate blocking and unblocking rates for wild-type Nav1.5 channels that we estimated by analyzing experimental whole-cell patch–clamp recordings using use-dependent block voltage protocols [1]:

For open-state block by cenobamate k_{ob}=0.00000215 nM^{-1}ms^{-1}, $k_{ob^{-1}}$=0.189 ms^{-1}, resulting in an open-state-specific $K_{d(o)} = k_{ob^{-1}}/ k_{ob}$ = 87,906.9767 nM;

For inactivated-state block by cenobamate k_{ib}=0.0000006698 nM^{-1}ms^{-1}, $k_{ib^{-1}}$= 0.18252 ms^{-1}, resulting in an inactivated-state-specific $K_{d(i)} = k_{ib^{-1}}/ k_{ib}$ = 272,499.2535 nM.

The ratio of the two K_d values was $K_{d(i)}/ K_{d(o)}$ = 3.099859, and we assumed that for the three Nav1.5 point mutants, this ratio should be preserved. We also assumed that, for mutant channels, only the unblocking rates change, while the state-specific blocking rates remain the same as for wild-type channels because a changed residue primarily modifies the interaction energy of the ligand to its modified binding site but not the energy barriers along the path of access to the blocking site. With these assumptions, which make quantitative rate estimates feasible, we obtained the following values for the three point mutants.

Mutant N1463K:

- Estimated $K_{d(o)}$ (by docking): 27.62 μM = 27,620 nM
- Estimated $K_{d(i)}$ = 3.099859 x $K_{d(o)}$ = 85,618.112 nM
- Eestimated state-specific unblocking rates:

$k_{ob^{-1}} = K_{d(o)} \times k_{ob}$ = 0.059383 ms^{-1} $k_{ib^{-1}} = K_{d(i)} \times k_{ib}$ = 0.057347 ms^{-1}

Mutant N1463Y:

- Estimated $K_{d(o)}$ (by docking): 38.613 μM = 38,130 nM
- Estimated $K_{d(i)}$ = 3.099859 x $K_{d(o)}$ = 118,197.633 nM
- Estimated state-specific unblocking rates:

$k_{ob^{-1}} = K_{d(o)} \times k_{ob}$ = 0.0819795 ms^{-1} $k_{ib^{-1}} = K_{d(i)} \times k_{ib}$ = 0.07916877 ms^{-1}

Mutant M1766R:

- Estimated $K_{d(o)}$ (by docking): 36.84 μM = 36,840 Nm
- Estimated $K_{d(i)}$ = 3.099859 x $K_{d(o)}$ = 114,198.814 nM
- Estimated state-specific unblocking rates:

$k_{ob^{-1}} = K_{d(o)} \times k_{ob}$ = 0.079206 ms^{-1} $k_{ib^{-1}} = K_{d(i)} \times k_{ib}$ = 0.076490 ms^{-1}
